# Involvement of Interleukin-10 in Analgesia of Electroacupuncture on Incision Pain

**DOI:** 10.1155/2019/8413576

**Published:** 2019-10-30

**Authors:** Wen-jing Dai, Jia-lu Sun, Chao Li, Wei Mao, Yun-ke Huang, Zhi-qi Zhao, Yu-qiu Zhang, Ning Lü

**Affiliations:** ^1^Department of Translational Neuroscience, Jing'an District Center Hospital of Shanghai, State Key Laboratory of Medical Neurobiology and MOE Frontiers Center for Brain Science and Institutes of Brain Science, Fudan University, Shanghai 200032, China; ^2^Institute of Acupuncture and Moxibustion, Fudan Institutes of Integrative Medicine, Shanghai 200032, China

## Abstract

**Objective:**

Postincision pain often occurs after surgery and is an emergency to be treated in clinic. Electroacupuncture (EA) is a Chinese traditional treatment widely used to cure acute or chronic pain, but its mechanism is not clear. Interleukin-10 (IL-10) is a powerful anti-inflammatory cytokine that shows neuroprotective effects in inflammation and injury in the CNS. The present study attempts to reveal that IL-10 is crucial for EA analgesia on postincision pain.

**Methods:**

A model of incision pain was established in C57BL/6J mice. The pain threshold was detected by behavioral test, and the expression of IL-10 and its receptor was detected by an immunohistochemical method. C-fiber-evoked field potentials were recorded by in vivo analysis.

**Results:**

The mechanical allodynia induced by paw incision was significantly inhibited by pretreatment of EA in mice. Intrathecal injection of IL-10 neutralizing antibody (2 *µ*g/10 *µ*L) but not intraplantar injection (10 *µ*g/10 *µ*L) reversed the analgesia of EA. The upregulations of IL-10 mRNA and protein were induced by EA at 6 h and 1 d after incision, respectively. Spinal long-term potentiation (LTP), a substrate for central sensitization, was also suppressed by EA with IL-10. IL-10 recombinant protein (1 *µ*g/10 *µ*L, i.t.) mimicked the analgesia of EA on mechanical allodynia and inhibition on the spinal LTP. Posttreatment of EA after incision also transitorily relieved the mechanical allodynia, which can be blocked by spinal IL-10 antibody. IL-10 and its receptor, IL-10RA, are predominantly expressed in the superficial spinal astrocytes.

**Conclusions:**

These results suggested that pretreatment of EA effectively prevented postincision pain and IL-10 in spinal astrocytes was critical for the analgesia of EA and central sensitization.

## 1. Introduction

Surgical injury is followed by pain, nausea, vomiting, paralytic ileus, risk of cardiopulmonary dysfunctions, and delayed convalescence [1]. Patients no longer tolerate prolonged suffering, and this may lead to demands for solutions that overcome complex problems [2]. The effect of early therapy for postoperative pain should be investigated because the intensity of acute postoperative pain is related to the risk of developing persistent pain [3–5]. Management of postoperative pain has been emphasized in several decades, but apparently with only small improvements.

Acupuncture, a traditional Chinese therapy, has been accepted to effectively treat various types of pain by inserting needles into specific acupuncture points on the patient's body [6, 7]. Evidence from meta-analyses shows that acupuncture appears to provide effective analgesia for some acute pain conditions in the emergency department, reducing acute postoperative pain, opioid requirements, and opioid adverse effects compared to controls [8]. Acupuncture is regarded as a low-cost, low-risk, limited minor adverse effects therapy.

Interleukin-10 (IL-10) is a multifunctional negative regulator, which is mainly produced by Th2 cells, activated B cells, monocytes, and macrophages. It is involved in the biological regulation of various cells, such as immune cells, inflammatory cells, and tumor cells [9, 10]. Moreover, IL-10 is produced by glial cells [11] and has an analgesic effect in inflammatory pain and neuropathic pain [12]. Infant nerve injury triggers an anti-inflammatory immune response, such as significant increases in IL-4 and IL-10, making a latent neuropathic pain emerge at adolescence [13]. Significant augmentation of severe spinal cord damages characterized by edema, tissue damage, and apoptosis on IL-10 KO mice indicated that IL-10 reduces the development of inflammation and tissue injury events associated with spinal cord trauma [14]. Thermal pain induced by neck incision was significantly suppressed by electroacupuncture (EA) on rats. Downregulation of substance P (SP), calcitonin gene-related peptide (CGRP), cyclooxygenase-1 (COX-1), and prostaglandin E2 (PGE2) induced by EA may contribute to the effects in relieving neck-incision pain [15]. It has been reported that manual acupuncture inhibited inflammatory cell infiltration and increased IL-10 levels in carrageenan-induced peritonitis in mice [16].

In the present study, we investigated whether IL-10 is involved in the analgesia of EA and postoperative pain.

## 2. Methods

### 2.1. Animal

Male C57BL/6J mice (25–30 g, *n* = 191) were supplied by Shanghai Experimental Animal Center of the Chinese Academy of Sciences. All mice were housed in a 12-h light/dark cycle at a room temperature of 22 ± 1°C and received food and water *ad libitum*. All animal experiments were approved by the Committee on the Use of Animal Experiments of Fudan University, and were in line with the policies issued by the International Association for the Study of Pain (IASP).

### 2.2. Electroacupuncture Treatment

Under light anesthesia with isoflurane (0.8–1.0% in oxygen) delivered via a nose cone, a pair of stainless steel pins of 0.16 mm diameter were inserted into the left Sanyinjiao (SP6) and Yanglingquan (GB34) acupoints on mice. SP6 is posterior to the mesial border of the tibia and 5 mm above the tip of the medial malleolus, and GB34 is located in the depression anteroinferior to the capitulum fibulae. The pins were inserted perpendicular to the skin at a depth of 2 mm (SP6) and 4 mm (GB34), respectively. The pin handles were connected to the HANS acupuncture point nerve stimulator (LH-202H, Huawei Co., Ltd., Beijing, China) for stimulating these acupoints with square wave current (pulse width: 0.2 ms, intensities ranging 1-2-3 mA and each intensity for 10 min, totaling 30 min) at 2 Hz and 100 Hz alternating frequencies (automatically shifting between 100 Hz and 2 Hz stimulation for 3 s each) [17]. Sham-EA control animals received needle insertion 2 or 4 mm above SP6 and GB34 (only pierced the skin) without EA stimulation.

### 2.3. Plantar Incision

All mice were anesthetized with 2% isoflurane, and a 7 mm longitudinal incision on the same side of acupuncture was made with a number 11 blade, starting 3 mm from the proximal edge of the heel to the toes along the midline of the plantar [18]. The plantaris muscle was elevated and incised longitudinally, leaving muscle origin and insertion intact. After hemostasis with gentle pressure, the wound was closed with 6-0 nylon suture. The mice were allowed to recover in their cages.

### 2.4. von Frey Test for Mechanical Allodynia

The mechanical threshold was measured by probing *von* Frey filaments (Stoelting, USA) on ipsilateral paws. Each mouse was placed on the elevated platform with 2 mm grids of iron wires throughout the entire area, and covered by 8 cm × 8 cm × 4 cm Plexiglas boxes. The mice were acclimated for at least 2 h each day, 2-3 days in advance, and for 30 min before testing. A series of *von* Frey filaments (0.16, 0.4, 0.6, 1.0, 1.4, and 2.0 g) were applied to the plantar surface of one hind paw. Each filament was tested 5 times with 15 s intervals. Paw withdrawal threshold (PWT) was defined as the lowest force that produced at least 3 withdrawal responses in 5 consecutive applications.

### 2.5. Drug Administration

Drugs were administered by lumbar puncture injection. Under isoflurane anesthesia, each mouse was placed on a Plexiglas tube to widen the intervertebral spaces [19]. No more than 10 *µ*l of drug was delivered into the spinal space with a 30-gauge needle between the L4 and L5 vertebrae. Mouse anti-IL-10 antibody (Santa Cruz, Sc-1783, USA), normal mouse IgG (Santa Cruz, Sc-2015, USA), or IL-10 recombination (GeneTex, GTX65349, USA) was injected over a period of 5 min. Sterile normal saline was used as the solvent control.

### 2.6. Western Blot

Mice were killed with overdoses of urethane, and the L4–L6 spinal cord segment was rapidly removed. The dorsal horn tissues were homogenized in lysis buffer containing a mixture of protease inhibitors and phenylmethylsulfonyl fluoride (Roche Diagnostics). Equal amount of protein was loaded and separated in 10% Tris-Tricine SDS-PAGE gel and transferred to PVDF membrane (Millipore). The membranes were blocked with 5% nonfat milk in Tris-buffered saline (pH 7.5) with 0.1% Tween-20 for 2 h at room temperature (RT) and incubated overnight at 4°C with goat anti-IL-10 antibody (1 : 250, R&D, AF519, USA), and goat anti-IL-10RA antibody (1 : 1000, R&D, AF-474-NA, USA). The blots were then incubated with HRP-conjugated secondary antibodies (1 : 2000, Pierce) for 2 h at 4°C. GAPDH antibody was probed as a loading control. Signals were finally detected using enhanced chemiluminescence (ECL, Thermo, USA), and the bands were visualized with the ChemiDoc XRS system (Bio-Rad, USA). All Western blot analysis was performed at least three times, and consistent results were obtained.

### 2.7. Quantitative RT-PCR

Tissues (the ipsilateral dorsal horns of L4–L6) were rapidly isolated in RNAse-free conditions and immediately transferred to TRIzol (Invitrogen). Total RNA was isolated following the manufacturer's instructions. cDNA was reverse transcribed with the PrimeScript™ RT reagent kit with gDNA Eraser (TAKARA, RR047A, Japan). RT-PCRs were performed using SYBR® Premix Ex Taq™ II (Tli RNase H Plus) (TAKARA, RR820W, Japan) and amplified in QuantStudio 3 (Thermo Fisher Scientific) using specific primer pairs for IL-10 (forward: 5′ ACAGCCGGGAAGACAATAAC 3′ and reverse: 5′ CAGCTGGTCCTTTGTTTGAAAG 3′) [20]; *β*-actin (forward: 5′ GGCTGTATTCCCCTCCATCG 3′ and reverse: 5′ GGCTGTATTCCCCTCCATCG 3′) (PrimerBank, http://pga.mgh.harvard.edu/primerbank/index.html) was used as the housekeeping gene. All PCRs were done in triplicate and the levels of *β*-actin were used to normalize results. The results were quantified using the ΔΔCT method.

### 2.8. Immunohistochemical Staining

After anesthetized with urethane (25%, 1.5 g/kg, i.p.), mice were transcardially perfused with 37°C saline followed by 4% paraformaldehyde. Thereafter, the L4–L6 spinal cord was taken, postfixed with 4% paraformaldehyde, and then dehydrated with a series of concentration gradient sucrose solutions (10%–20%–30%) in PB for 24–48 h at 4°C. The spinal cord tissues were transected into 35 *µ*m sections with a freezing microtome (Leica, Germany). The sections were first blocked by 10% normal donkey serum in 0.3% Triton X-100 for 1 h at room temperature and then incubated with a mixture of goat anti-IL-10 (1 : 200, R&D System, AF519, USA), rabbit anti-IL-10 RA (1 : 500, Abcam, Ab225820, UK), rabbit anti-Iba-1 (1 : 500, Wako Pure Chemical Industries, 019-19741, Japan), goat anti-Iba-1 (1 : 500, Abcam, ab5076, UK), mouse anti-GFAP (1 : 2000, Sigma-Aldrich, G6171, USA), mouse anti-NeuN (1 : 2000, Millipore, MAB377, USA), or mouse anti-*β*3-tubulin (1 : 500, Covance, MMS-435P, USA) overnight at 4°C. After rinsing, the sections were incubated with donkey anti-goat Alexa Flour 488 (1 : 200, Invitrogen, A-11055, USA), donkey anti-rabbit Alexa Flour 546 (1 : 200, Invitrogen, A-10040, USA), or donkey anti-mouse Alexa Flour 546 (1 : 200, Invitrogen, A-10036, USA) under the dark condition for 2 h at room temperature. The stained sections were examined by a confocal laser-scanning microscope (FV1000; Olympus, Tokyo, Japan).

### 2.9. Electrophysiological Recording of Spinal LTP

The electrophysiological experiments were performed according to the previous study [21]. Mice were anesthetized with 25% urethane (1.5 g/kg, i.p.). The trachea was cannulated to allow mechanical ventilation with room air, if necessary. A laminectomy was performed at vertebrae T13 to L1 to expose the lumbar enlargement. An intrathecal catheter (PE-10 tube) was inserted through the gap between the L4 and L5 vertebrae and extended to the subarachniod space of the lumbar enlargement. The catheter was filled with sterile normal saline (NS, approximately 2 *µ*l) and the outer end was plugged. Drug or vehicle was injected over a period of 1 min via the catheter at a volume of 10 *µ*l, followed by 2 *µ*l NS for flushing [17]. The left sciatic nerve was dissected free to deliver electrical stimulation with bipolar silver electrodes and covered with warm paraffin oil. Colorectal temperature was kept constant at 37 ± 0.5°C by a feedback-controlled heating blanket.

Following electrical stimulation of the sciatic nerve, the field potentials were recorded in the ipsilateral L4-L5 spinal cord segments with glass microelectrodes (3∼5 MΩ impedance), 100–300 *µ*m from the surface of the cord. After recording stable responses following test stimuli (2 × C-fiber threshold, 0.5 ms, 60 s interval) for more than 30 min, conditioning tetanic stimulation (4 × C-fiber threshold, 100 Hz, 0.5 ms, 4 trains of 1 s duration at 10 s interval) was delivered to the sciatic nerve for induced LTP of C-fiber-evoked field potentials. As a control, the sham group was not applied with conditioning tetanic stimulation. The signals were amplified and converted into a digital signal, respectively, by a microelectrode AC amplifier (A-M System, USA) and CED systems (A/D converter Micro 1401 mkII;, recording software Spike 2, CED, UK).

The amplitudes of C-fiber-evoked field potentials were determined offline by Spike 2 version 6. In each experiment, responses to 10 consecutive test stimuli were averaged. Data were normalized to mean values of the first 30-min amplitudes of C-fiber-evoked field potentials.

### 2.10. Statistical Analysis

Data were expressed as mean ± SEM. Student's *t*-test, one-way, or two-way ANOVA followed by post hoc Student–Newmann–Keuls test was used to identify significant difference. In all cases, *P* < 0.05 was considered as statistically significant.

## 3. Results

### 3.1. IL-10 Is Involved in Analgesia of Electroacupuncture on Incision Pain

Consistent with our previous study [22], the surgical incision applied on the hind paw induced a robust mechanical allodynia in mice lasting one week. Pretreatment of EA significantly inhibited the mechanical allodynia induced by the incision.

**Figure 1:**
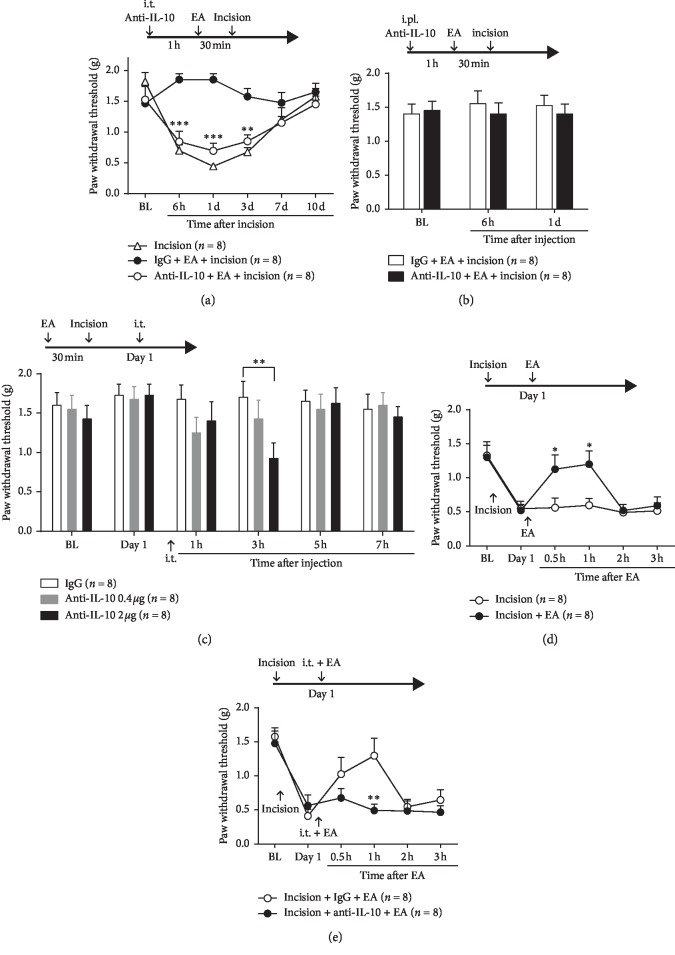
Involvement of IL-10 in the analgesia of electroacupuncture (EA) on incision pain. (a) Incision-induced mechanical allodynia was blocked by EA (2/100 Hz, 1-2-3 mA, 30 min) and the analgesia effect of EA was reversed by lumbar puncture injection of anti-IL-10 neutralizing antibody (2 *µ*g/10 *µ*L) delivered 1 h before EA (*P* < 0.001, vs. IgG). (b) The analgesic effect of EA was not inhibited by intraplantar injection of anti-IL-10 antibody (10 *µ*g/10 *µ*L) 1 h before EA (*P* > 0.05, vs. IgG). (c) IL-10 neutralizing antibody (0.4 *µ*g or 2 *µ*g) was intrathecally injected on 1 d after incision and the ipsilateral PWTs were dose-dependently decreased at 3 h after injection compared with control IgG (*P* < 0.01, vs. IgG). (d) The incision-induced mechanical allodynia was relieved at 0.5 and 1 h after EA performed at 1 d after incision compared with the incision group (*P* < 0.01, vs. incision). (e) The analgesic effect at 1 h after EA was significantly blocked by intrathecal injection of IL-10 antibody 1 h before EA (*P* < 0.01, vs. IgG). ^*∗*^*P* < 0.05, ^*∗∗*^*P* < 0.01, ^*∗∗∗*^*P* < 0.001.

To address whether spinal IL-10 is involved in the analgesia of EA, we examined the influence of blocking IL-10 on incision-induced allodynia. IL-10 neutralizing antibody (2 *µ*g/10 *µ*L) was delivered intrathecally 1 h before EA, and the plantar incision was performed immediately after EA (2/100 Hz, 1-2-3 mA, 30 min). The mechanical threshold decreased significantly at 6 h, 1 d, and 3 d after incision compared with that in the IgG group (Figure 1(a); two-way ANOVA, treatments × time: *F*_10,70_ = 7.457, *P* < 0.001).

Interestingly, the analgesia of EA was not affected by intraplantar injection of IL-10 neutralizing antibody (10 *µ*g/10 *µ*L) 1 h before EA. The ipsilateral PWTs for *von* Frey filaments did not decrease (Figure 1(b), two-way ANOVA, treatments × time: *F*_2,14_ = 0.2698, *P* > 0.05).

To confirm the role of IL-10 in the analgesia effect of EA pretreatment, IL-10 neutralizing antibody (0.4 *µ*g or 2 *µ*g) intrathecally injected on 1 d after incision dose-relatedly decreased the ipsilateral PWTs in the *von* Frey test. Mechanical allodynia was obviously induced at 3 h after injection only in the dose of 2 *µ*g compared with control IgG (Figure 1(c); two-way ANOVA, treatments × time: *F*_10,70_ = 0.9938, *P* < 0.01).

According to previous reports, EA relieved inflammatory pain and neuropathic pain [23, 24]. In this study, EA was also performed at 1 d after incision and the mechanical allodynia was ameliorated at 0.5 and 1 h after EA compared with that in the incision group, in which no EA was performed after incision (Figure 1(d); two-way ANOVA, treatments × time: *F*_5,35_ = 2.437, *P* < 0.01). Moreover, the analgesic effect at 1 h after EA was significantly blocked by intrathecal injection of IL-10 antibody 1 h prior to EA (Figure 1(e); two-way ANOVA, treatments × time: *F*_5,35_ = 2.978, *P* < 0.01).

### 3.2. EA Upregulates IL-10 Gene or Protein Expression

**Figure 2:**
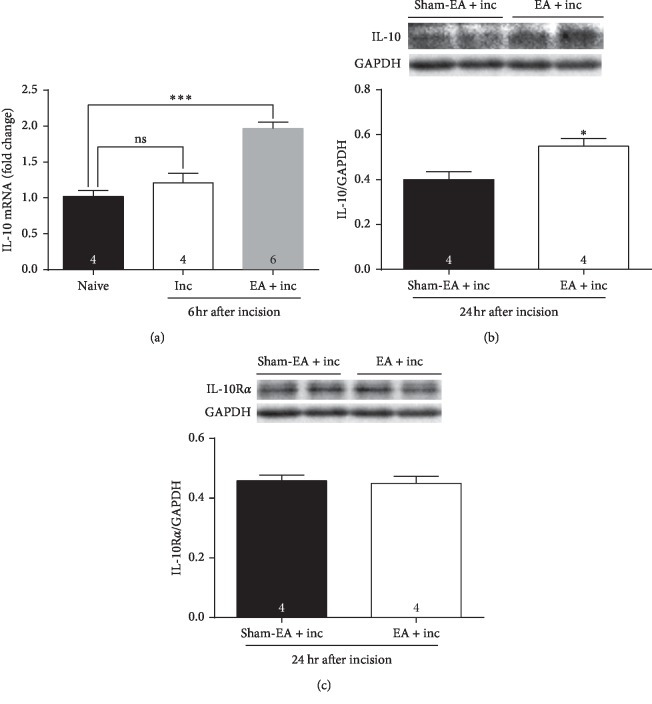
IL-10 was upregulated by EA. (a) IL-10 mRNA was not increased by incision (*P* > 0.05, vs. naïve), but significantly increased by pretreatment of EA at 6 h after incision (*P* < 0.001, vs. naïve). (b) IL-10 protein expression in the EA group was significantly higher than that in the sham-EA group at 1 d after incision (*P* < 0.05, vs. sham-EA + inc). (c) IL-10RA was not different between the two groups (*P* > 0.05, vs. sham-EA + inc).

To detect whether IL-10 and IL-10RA could be affected by incision or pretreatment of EA, the mRNA of IL-10 was quantified at 6 h after incision in groups of naïve, incision, and EA + incision. Data showed that IL-10 mRNA was not increased in the incision group compared with naïve mice, but significantly increased in the EA + incision group (Figure 2(a), one-way ANOVA, treatments: *F*_2,10_ = 24.64, *P* < 0.001).

The protein expression of spinal IL-10 and IL-10RA was quantified in the EA + incision group and the sham-EA + incision group at 1 d after incision. Western blot analysis showed significant upregulation of IL-10 in the EA group compared with the sham-EA group (Figure 2(b), Student's *t*-test, *P* < 0.05). IL-10RA protein expression was not different between the two groups (Figure 2(c), Student's *t*-test, *P* > 0.05).

### 3.3. IL-10 Contributes to Suppression of EA on Spinal LTP

**Figure 3:**
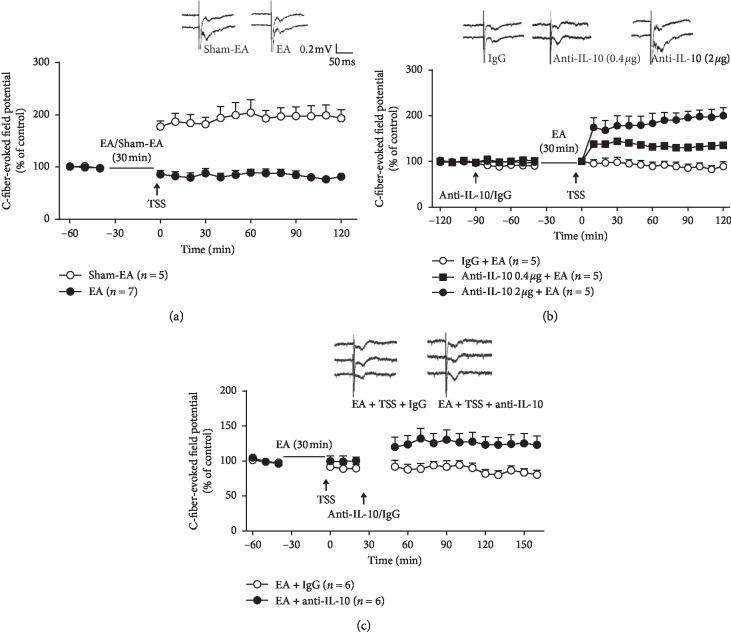
Involvement of IL-10 in the inhibition of EA on spinal LTP. (a) The spinal LTP completely blocked by EA performed 30 min before the tetanic stimulation of the sciatic nerve (TSS) compared to sham-EA (*P* < 0.001, vs. sham-EA). (b) The IL-10 antibody (0.4 *µ*g or 2 *µ*g) recovered the spinal LTP suppressed by EA in a dose-dependent manner (*P* < 0.001, vs. IgG). (c) The spinal LTP was also recovered by IL-10 neutralizing antibody (2 *µ*g) intrathecally injected 30 min after TSS (*P* < 0.05, vs. IgG).

Previous studies showed that LTP of C-fiber-evoked potentials in the spinal cord was regarded as a substrate for central sensitization of the pain pathway which amplifies nociceptive input and results in hyperalgesia [25, 26]. In this study, the spinal LTP significantly alleviated by EA performed 30 min earlier than the tetanic stimulation of the sciatic nerve (TSS) compared to sham-EA (Figure 3(a); two-way ANOVA, treatments: *F*_1,160_ = 486.6, *P* < 0.001).

To verify whether IL-10 was involved in the depression of EA on spinal LTP, IL-10 neutralizing antibody was delivered intrathecally by the PE-10 tube 1 h before EA. The IL-10 antibody (0.4 *µ*g or 2 *µ*g) dose-relatedly recovered the LTP which was blocked by EA in the IgG group (Figure 3(b); two-way ANOVA, treatments: *F*_2,264_ = 168.8, *P* < 0.001).

In addition, the spinal LTP was also recovered when IL-10 neutralizing antibody (2 *µ*g) was intrathecally injected 30 min after the inhibition of spinal LTP induced by EA (Figure 3(c); two-way ANOVA, treatments: *F*_1,177_ = 79.84, *P* < 0.05).

### 3.4. Exogenetic IL-10 Mimics the Analgesic Effect of EA

**Figure 4:**
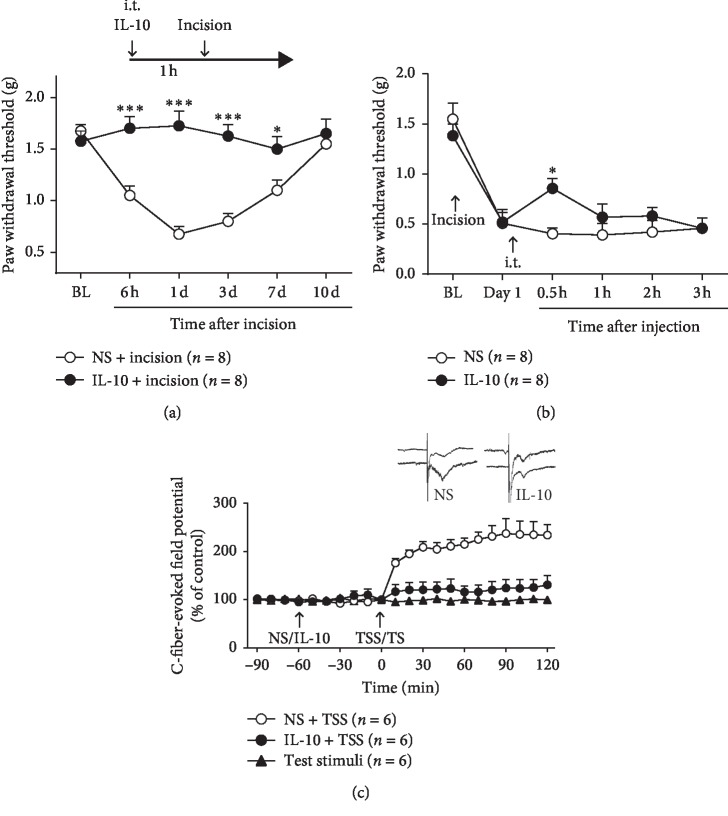
Exogenous IL-10 mimicked the analgesic effect of EA. (a) The mechanical allodynia was relieved by IL-10 recombinant protein (1 *µ*g/10 *µ*L) intrathecally injected 1 h before incision (*P* < 0.001, vs. vehicle). (b) Intrathecal injection of exogenetic IL-10 (1 *µ*g/10 *µ*L) at 1 d after incision alleviated the mechanical allodynia at 0.5 h after injection (*P* < 0.01, vs. vehicle). (c) The spinal LTP was also blocked by IL-10 recombinant protein (1 *µ*g/10 *µ*L) injected into the spinal subarachnoid space 1 h before TSS (*P* < 0.001, vs. vehicle). ^*∗*^*P* < 0.05, ^*∗∗∗*^*P* < 0.001.

To further address the involvement of IL-10 in the analgesia effect of EA, we inspected the effects of the IL-10 recombinant protein on the incision-induced allodynia and spinal LTP. The mechanical allodynia was attenuated by the IL-10 recombinant protein (1 *µ*g/10 *µ*L) intrathecally injected 1 h before incision (Figure 4(a); two-way ANOVA, treatments × time: *F*_5,35_ = 7.912, *P* < 0.001).

Likewise, a single intrathecal injection of exogenous IL-10 (1 *µ*g/10 *µ*L) at 1 d after incision alleviated the mechanical allodynia at 0.5 h after the injection (Figure 4(b); two-way ANOVA, treatments × time: *F*_5,35_ = 1.791, *P* < 0.01).

Additionally, the spinal LTP was also blocked by IL-10 recombinant protein (1 *µ*g/10 *µ*L) injected into the spinal subarachnoid space similar to EA compared with the vehicle (Figure 4(c), two-way ANOVA, treatments: *F*_2,330_ = 197.9, *P* < 0.001).

### 3.5. Expression of IL-10 and IL-10RA in the Spinal Cord

**Figure 5:**
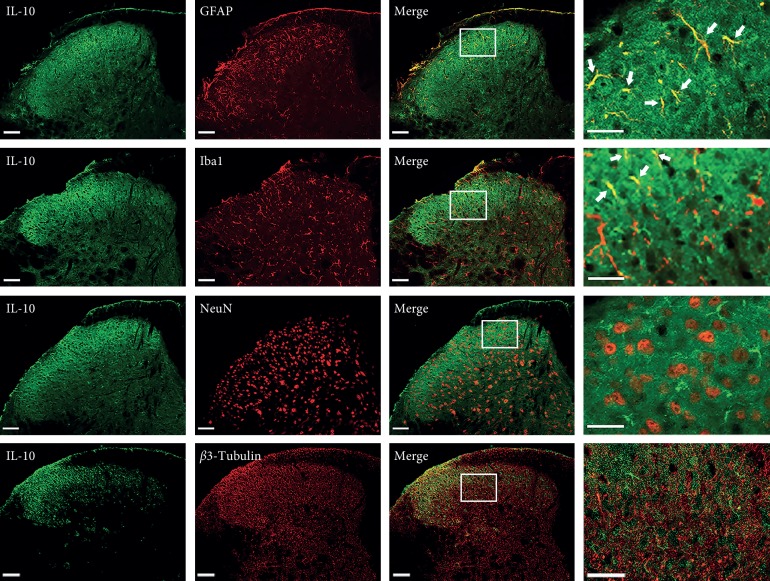
Expression of IL-10 in the spinal cord. Double immunofluorescence revealed that IL-10 was mainly expressed in GFAP-labeled astrocytes, slightly expressed in Iba-1-labeled microglia in normal mice but seldom in NeuN or *β*3-tubulin-labeled neurons (scale bar, 50 *µ*m).

**Figure 6:**
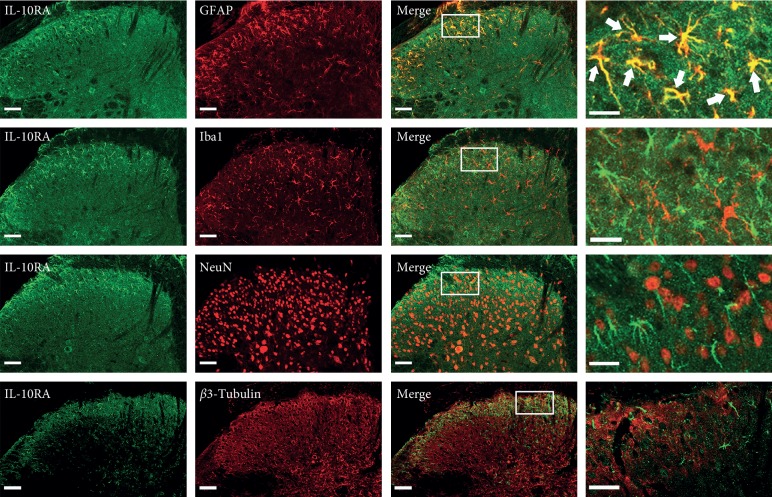
Expression of IL-10RA in the spinal cord. IL-10RA was primarily expressed in GFAP-labeled astrocytes but not in NeuN- or *β*3-tubulin-labeled neurons and Iba-1-labeled microglia (scale bar, 50 *µ*m).

To determine the spatial distribution of IL-10 and IL-10RA, we performed double immunofluorescent staining for IL-10/IL-10RA and NeuN/*β*3-tubulin (neuronal markers), Iba-1 (a microglial marker), or GFAP (an astrocytic marker) on sections of the L4–L6 spinal cord. Moderate IL-10/IL-10RA immunoreactivity was found in superficial layers I-II of the dorsal horn of normal mice, and was mainly colocalized with GFAP. IL-10 was slightly coexpressed with Iba-1. Both IL-10- and IL-10RA-labeled cells were rarely found in NeuN- or *β*3-tubulin-labeled neurons (Figures 5–6).

## 4. Discussion

The present study revealed an involvement of IL-10 in analgesia of EA on incision pain. Pretreatment EA significantly blocked mechanical allodynia induced by paw incision, while EA performed after incision relieved mechanical allodynia transitorily. IL-10 was increased in the spinal dorsal horn by pretreatment of EA. IL-10 and its receptor, IL-10RA, were mainly expressed in the superficial spinal astrocytes. IL-10 in the spinal cord but not in the peripheral tissue was necessary for the analgesic effect of EA and central sensitization.

Many reports have shown that most surgical procedures may be followed by a risk of persistent pain syndromes. Patients undergo surgery for different conditions, and about 10 to 50% of them develop persistent postsurgery pain [1]. A systematic review on trials including several types of surgery has showed that different acupuncture techniques may be effective for the alleviation of postoperative pain. Opioid consumption was significantly reduced, as well as the incidence of opioid-related adverse events [27]. Lan et al. investigated the effect of transcutaneous electrical nerve stimulation (TENS) at P6, LI4, ST36, and GB31 after total hip arthroplastry. Patients in the TENS group received electrical stimulation before and after surgery. The data showed that there was no difference in pain intensity between the TENS group and sham (no stimulation of the electrodes). However, they found that the acustimulation group required less opioids and analgesics and experienced less nausea and vomiting [28].

A study was performed on 18 dogs for ovariohysterectomy. Three groups received EA through needles inserted at ST36, SP6 and GB34, or at peri-incisional dermatomes (DER) [18], or as a combination of the two (EAD). The stimulation was applied before induction of anesthesia. The trial indicated that EA and EAD groups had significantly lower pain scores at the first hours postoperatively compared with the DER group. However, after three hours, the differences were negligible. The lack of a difference in pain scores among the treatment groups after 3 h postoperatively was because more than 80% of the dogs in the DER group received supplemental morphine, in contrast to 33% of the dogs in the EA and EAD groups [29]. In this study, mechanical allodynia was expressively induced by paw incision. EA performed before incision significantly blocked the postoperative pain lasting several days. On the other hand, EA performed after incision only partially relieved mechanical allodynia within one hour, according to previous reports.

Acupuncture is strikingly effective for relieving incision pain, but its mechanism of analgesia is not clear. Chronic pain is maintained in part by central sensitization, a phenomenon of synaptic plasticity, and increased neuronal responsiveness in central pain pathways after painful insults [30]. Spinal LTP is an important form of synaptic plasticity and a unique form of central sensitization in chronic pain. There are striking similarities between spinal LTP and central sensitization, and both show the critical requirements of NMDA receptor and involvement of key signaling transduction pathways, including the protein kinase C, extracellular signal-regulated kinase, and Src, as well as dependence of protein synthesis and gene transcription. Our data showed that EA arranged before tetanic stimuli on the sciatic nerve markedly inhibited spinal LTP. Acupuncture may suppress the central sensitization to relieve incision pain.

IL-10 exerts a wide spectrum of regulatory activities in the immune and inflammatory response and even in cancer. Recent work showing IL-10 production from regulatory T cells and even T-helper 1 T cells has revealed the power of this cytokine to influence immune responses. The binding of IL-10 to the receptor complex activates JAK1 and Tyk2, associated with IL-10RA and IL-10RB, respectively, to phosphorylate the cytoplasmic tails of the receptors. This results in the recruitment of STAT3 to the IL-10RA (B) [31]. It was reported that the antinociceptive effects of gabapentin on morphine might be caused by activation of the IL-10 signaling pathway, which resulted in the inhibition of the expression of pro-inflammatory cytokines in neuropathic pain in the rat spinal cord [32]. The IL4-10 fusion protein was more effective in inhibiting allodynia in a mouse model of neuropathic pain and the activity of glial cells [33]. Recombinant rat IL-10 not only reduced the densities of TTX-sensitive and Nav1.8 currents in control DRG neurons, but also reversed the increase of sodium currents induced by rat recombinant TNF-*α*, suggesting that the downregulation of the sodium channels in DRG neurons might contribute to the therapeutic effect of IL-10 on neuropathic pain [34]. SP6 manual acupuncture increased muscle IL-10 levels and was ineffective in reducing pain behaviors and edema in IL-10 knockout (IL-10−/−) mice [35]. The results of our experiments showed that IL-10 neutralized antibody notably reversed the analgesia of EA and the inhibition of spinal LTP, indicating that IL-10 played a crucial role in it.

A recent report characterized IL-10RA in embryonic spinal cord neurons; following transgene IL-10 stimulation in neuronal cell culture, postnatal IL-10RA is expressed in astrocytes, microglia/perivascular microglia, oligodendrocytes, and endothelial cells, but not neurons, in the intact brain [36]. In the adult brain and spinal cord, the cellular pattern of IL-10RA expression is similar to that observed in the immature postnatal central nervous system, with expression observed in astrocytes, microglia/macrophages, and oligodendrocytes under pathological conditions. In a separate study, the spinal distribution of IL-10 was also reported in non-neuronal cells [37]. In the present study, IL-10 and IL-10RA mainly distributed in the superficial spinal cord and colocalized with the astrocyte marker GFAP, suggesting that IL-10 controlled glial proinflammatory products that act to enhance pain transmission by autocrine and paracrine pathways.

## 5. Conclusion

These results suggested that pretreatment of EA effectively prevented postincision pain and IL-10 in spinal astrocytes was critical for the analgesia of EA and central sensitization.

## Data Availability

The data used to support the findings of this study are available from the corresponding author upon request.
